# Twenty-four-hour real-time continuous monitoring of acute focal cerebral ischemia in rabbits based on magnetic inductive phase shift

**DOI:** 10.1186/s12938-020-00829-5

**Published:** 2020-11-11

**Authors:** Shuang-Lin Zhao, Gui Jin, Ze-Lin Bai, Jing-Bo Chen, Meng-Wei Li, Gen Li, Wei Zhuang, Yue-Ning Liu, Ming-Xin Qin

**Affiliations:** 1 College of Biomedical Engineering, Army Medical University, Chongqing, 400038 China; 2 Department of Medical Engineering, Beidaihe Rehabilitation and Recuperation Center, Hebei, 066100 China; 3 School of Pharmacy and Bioengineering, Chongqing University of Technology, Chongqing, 400020 China

**Keywords:** Cerebral ischemic stroke, Magnetic induction phase shift, Thrombin induction method, Symmetric cancellation-type magnetic induction sensor

## Abstract

**Background:**

As a serious clinical disease, ischemic stroke is usually detected through magnetic resonance imaging and computed tomography. In this study, a noninvasive, non-contact, real-time continuous monitoring system was constructed on the basis of magnetic induction phase shift (MIPS) technology. The “thrombin induction method”, which conformed to the clinical pathological development process of ischemic stroke, was used to construct an acute focal cerebral ischemia model of rabbits. In the MIPS measurement, a “symmetric cancellation-type” magnetic induction sensor was used to improve the sensitivity and antijamming capability of phase detection.

**Methods:**

A 24-h MIPS monitoring experiment was carried out on 15 rabbits (10 in the experimental group and five in the control group). Brain tissues were taken from seven rabbits for the 2% triphenyl tetrazolium chloride staining and verification of the animal model.

**Results:**

The nonparametric independent-sample Wilcoxon rank sum test showed significant differences (*p* < 0.05) between the experimental group and the control group in MIPS. Results showed that the rabbit MIPS presented a declining trend at first and then an increasing trend in the experimental group, which may reflect the pathological development process of cerebral ischemic stroke. Moreover, TTC staining results showed that the focal cerebral infarction area increased with the development of time

**Conclusions:**

Our experimental study indicated that the MIPS technology has a potential ability of differentiating the development process of cytotoxic edema from that of vasogenic edema, both of which are caused by cerebral ischemia.

## Background

Most cerebral stroke cases are cerebral infarction caused by temporary or permanent cerebrovascular occlusion [1]. Cerebral ischemic stroke is characterized by complex pathogenesis and high fatality rate and disability rate, and it tends to attack at the earlier age in recent years. Global stroke burden is mainly concentrated in low-income and medium-income countries [2]. Cerebral edema is a common secondary disease of cerebral stroke, and evidence showed that cerebral edema is an independent predictive factor for prognosis of patients with stroke [3, 4]. At the initial development stage of ischemic brain injury, cytotoxic edema occurs in the ischemic region with the rapid development of irreversible injury [5]. Vasogenic edema is also formed because of the damage in the blood–brain barrier (BBB), thus leading to tissue swelling [6]. Real-time continuous monitoring of cerebral edema is highly important for observing the state of disease of patients with cerebral stroke, guiding the treatment, determining the operation opportunity, and evaluating the prognosis. The commonly used detection methods for patients with cerebral ischemic stroke mainly include computed tomography (CT), magnetic resonance imaging (MRI), intracranial pressure (ICP) detection, transcranial Doppler sonography (TCD), and electrical bioimpedance (EBI) technology. At the early stage of ischemia, continuous CT and ICP monitoring results help identify high-risk patients with obvious brain swelling [7]. However, as invasive monitoring, ICP results in the risk of bleeding and infection. Although CT and MRI do not lead to complications, the equipment cost is high [8], real-time detection is impossible [9], and they cannot easily detect early acute cerebral ischemia, which may delay diagnosis [10, 11]. TCD could not monitor edema accurately enough [9]. As for EBI technology, the electrode contacts the epidermis, and due to high electrical resistivity of the skull, the injection current experience attenuations with poor penetrability, which seriously affects the measurement accuracy [12]. Therefore, a noninvasive, non-contact, bedside real-time monitoring system is urgently and clinically needed.

Magnetic induction phase shift (MIPS) technology is a method that could realize noninvasive, non-contact, real-time continuous monitoring. It is based on electrical conductivity and dielectric constant of the measured object. This method has been applied to studies on cerebral ischemia, cerebral edema, cerebral hemorrhage, and cerebral trauma [8, 13–22]. As for experimental studies on cerebral ischemia, Gonzalez et al. [19] constructed an acute focal cerebral ischemia model by combining ligation of the right common carotid artery (CCA) and electrocoagulation of the middle cerebral artery (MCA) in mice after craniotomy. Wei Zhuang et al. [8] established a whole-brain ischemia model through ligation of bilateral CCA and monitored this model for 1 h on the basis of MIPS technology. Shuanglin Zhao et al. [23] constructed a rabbit whole-brain edema model by using epidural freezing-induced cerebral edema. “Coaxial two-coil” sensors were used in all of the above-mentioned experimental studies.

In the present study, an MIPS monitoring system was constructed for noninvasive, non-contact, real-time continuous monitoring and an experimental research on 24-h monitoring of acute focal cerebral ischemia in rabbits was carried out to investigate the feasibility of using MIPS technology to detect acute focal cerebral ischemia. In the experimental research, the acute focal cerebral ischemia model in rabbits established using the “thrombin induction method” [24] was taken as the research object to reduce cerebral trauma in the animal experiment and make the animal model more agreeable with the clinical pathological development process of cerebral ischemic stroke. A self-made “symmetric cancellation-type” magnetic induction sensor was used in the experiment to improve the detection sensitivity and eliminate the disturbances from environmental electromagnetic field and normal physiological changes in rabbits.

## Results

### TTC staining results

**Fig. 1.**
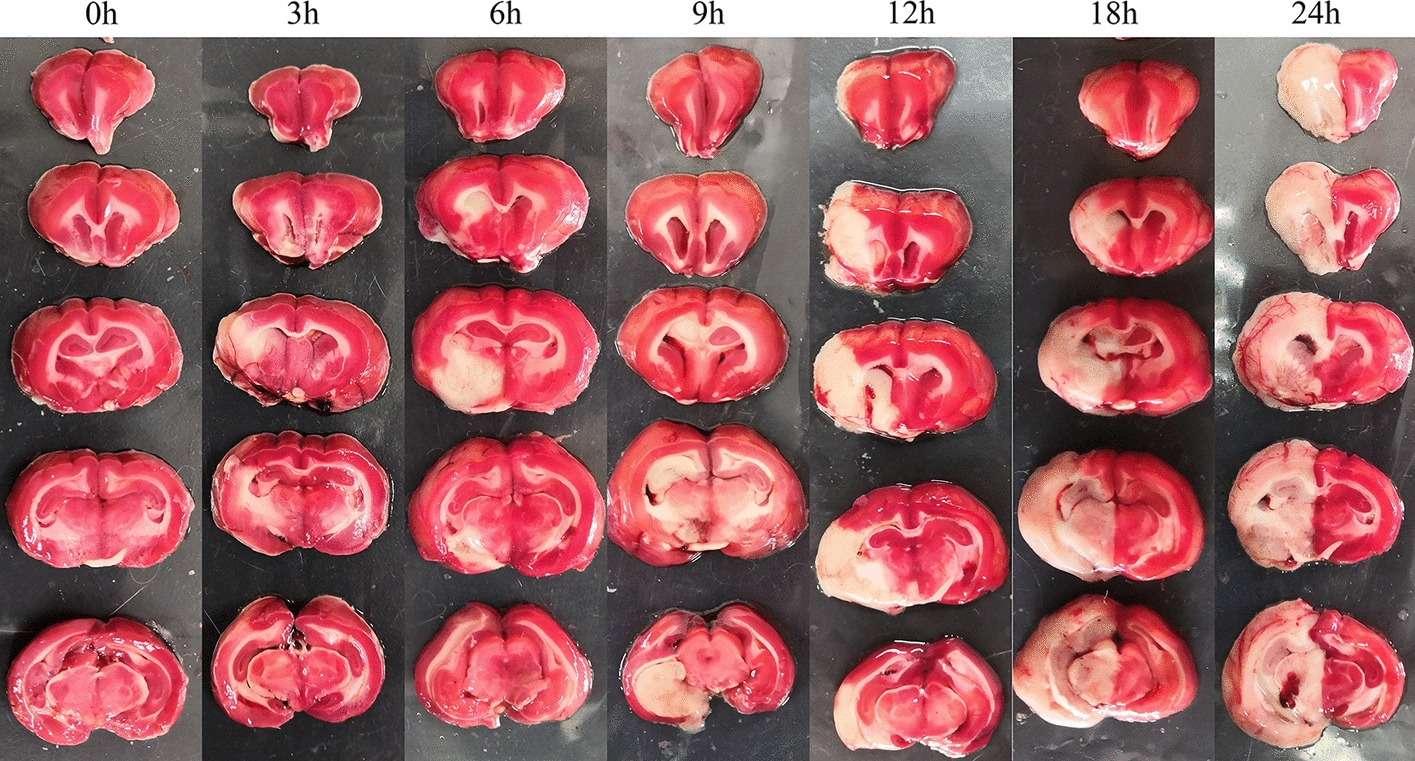
2% TTC staining results at 0, 3, 6, 9, 12, 18, and 24 h after modeling

Figure 1 shows the 2% TTC staining images of 16–22# rabbits; the rabbit brains were taken at 0, 3, 6, 9, 12, 18, and 24 h after modeling, respectively, for staining. Infarction did not appear on the rabbit brain sections at 0 h. As the time passed by, the infarcted region gradually expanded. At 24 h, swelling could be obviously observed in the left-brain hemisphere, indicating that the established animal model was successful and effective. As time went on, the focal cerebral ischemia in the rabbits worsened and the injury area enlarged.

### MIPS measurement results

**Fig. 2:**
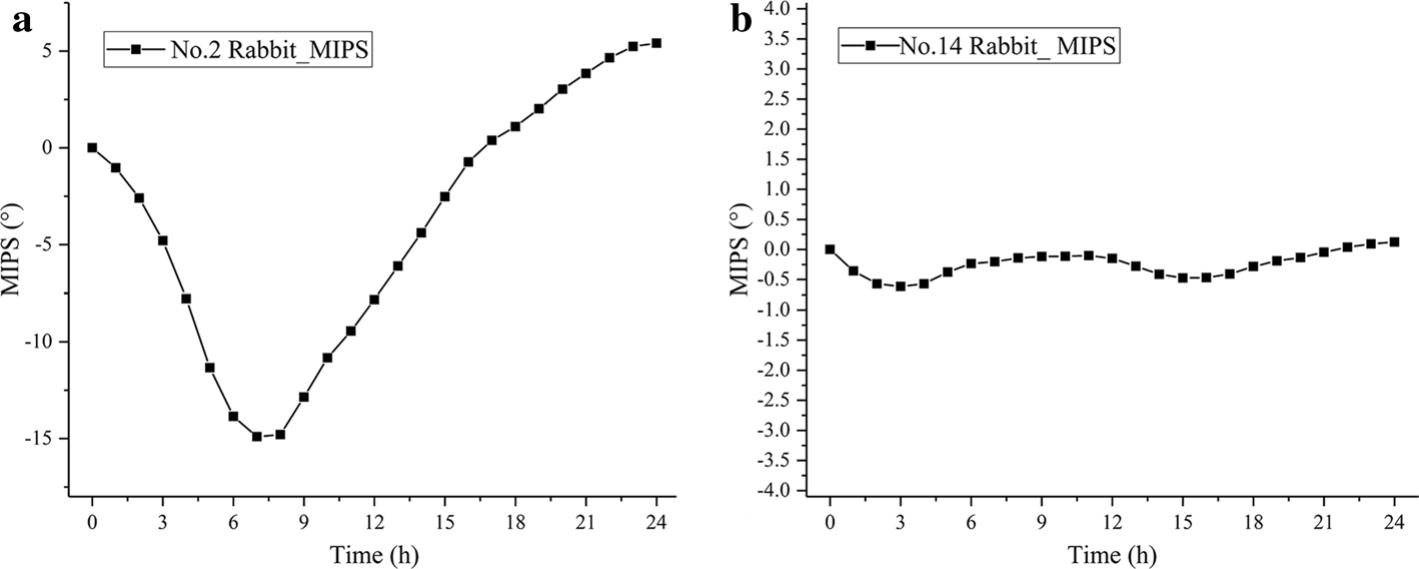
**a** MIPS variation trend of 2# and **b** 14# rabbits within 24 h

Figure 2a, b displays the MIPS measurement results of 2# and 14# rabbits at their characteristic frequency points of 82.02 MHz and 87.71 MHz, respectively. Figure 2a presents the MIPS variation trend of 2# rabbit in the experimental group within 24 h. It first declined to a minimum point of − 14.91 at 7 h and then increased. Figure 2b shows the MIPS variation of 14# rabbit in the control group with 24 h. No evident increase nor decrease occurred, and the average variation of the MIPS data was − 0.24° ± 0.21° within 24 h.

**Fig. 3:**
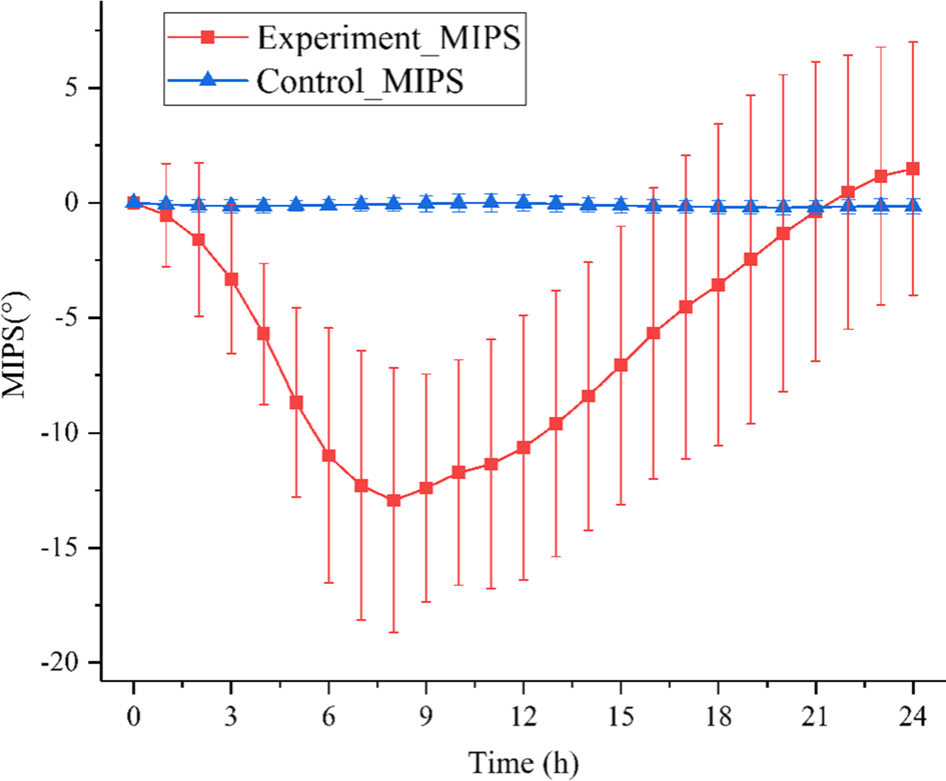
Variation trends of MIPS mean ± standard deviation of the experimental group (*n* = 10) and control group (*n* = 5) within 24 h

Figure 3 displays the variation trends of MIPS mean in the experimental group (n = 10) and control group (n = 5) at characteristic frequency points of 84.49 ± 1.48 and 86.05 ± 2.04 MHz within 24 h. The MIPS mean also first declined to a minimum point of − 12.93° ± 5.74° at 8 h and then increased in the experimental group. The MIPS variation trend in the experimental group was ascribed to the first decreasing and then increasing trends of electrical conductivity under the occurrence of focal cerebral ischemia [19, 25]. The MIPS mean of the control group did not experience any obvious increase nor decrease, and its average variation was − 0.11° ± 0.28° within 24 h.

**Fig. 4:**
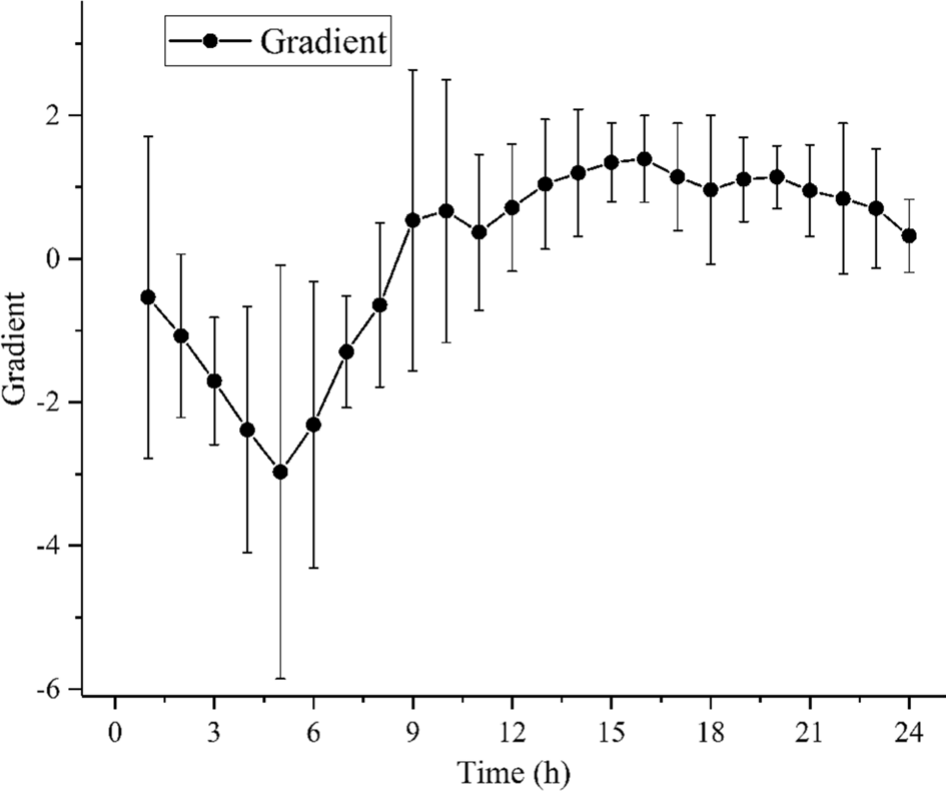
Mean ± standard deviation of MIPS variation rate in the experimental group (n = 10)

The MIPS variation rate in the experimental group is shown in Fig. 4. The MIPS rapidly declined in the first 5 h. This decline slowed down after 5 h, and the MIPS started to increase reversely at 9 h. After 16 h, the increase in MIPS tended to be steady. This finding indicated that the brain conductivity declined rapidly in the first 5 h, the declining rate was reduced after 5 h, and it started rising at 9 h. In addition, 5 and 16 h were the times when the electrical conductivity declined and rapidly increased the most.

### Statistical results

Nonparametric two-independent-sample rank sum test was carried out on the MIPS data of the experimental group and the control group, and the test results are listed in Table 1. The Z-statistic was − 4.618 (*p* = 0.000 < 0.05). Thus, the difference was statistically significant. In addition, a significant difference was found in the MIPS data between the experimental group and the control group. This difference was caused by focal cerebral ischemia, indicating that this system was feasible and effective when used to monitor acute focal cerebral ischemia in rabbits.

**Table 1:** Nonparametric two-independent-sample rank sum test results of the experimental group and control group

Mann–Whitney U	Wilcoxon W	Z	Asymp. Sig. (two-tailed)
74.500	399.500	− 4.618	0.000

The results of the nonparametric two-independent-sample rank sum test of the MIPS data at the initial time and those at 1 and 2 h in the experimental group were statistically analyzed, as seen in Table 2. At 1 h, *p* = 0.095 > 0.05 in the experimental group, indicating that the MIPS data at 1 h did not statistically differ from that at the initial time in the experimental group. The p value started to become smaller than 0.05 from 2 h, and the difference was statistically significant. This finding suggested that before 1 h, no obvious cerebral ischemia was induced, possibly because thrombin did not exert a complete effect in the blood vessels after injection. After 1 h, the thrombin-induced thrombus seriously blocked the blood vessels and then caused changes in the MIPS data.

**Table 2:** Nonparametric two-independent-sample rank sum test results of MIPS data in the experimental group at 1 and 2 h and that at initial time

Time	Sample	Mann–Whitney U	Wilcoxon W	Z	Asymp. Sig. (two-tailed)
1 h	Experiment	40.000	95.000	− 0.808	0.419
2 h	Experiment	20.000	75.000	− 2.423	0.015

## Discussion

In this study, an acute focal cerebral ischemia model in rabbits was constructed using the “thrombin induction method” [24]. This model showed a higher success rate than the traditional model established through the “intraluminal thread technique”. Moreover, it triggered smaller trauma and more conformed to the clinical pathological development process of patients with cerebral stroke than the rabbit autologous blood-injected cerebral hemorrhage model [26], the bilateral carotid artery ligation-induced cerebral ischemia model [8], and the epidural freezing-induced cerebral edema model [9] previously established. By injecting thrombin in the ICA, the blood was naturally coagulated in MCA to form thrombus, which blocks the MCA.

The structure of the “symmetric cancellation-type” magnetic induction sensor improved compared with the “symmetric cancellation-type” sensor designed by Jin et al. [26]. The object was placed between the excitation coil and the detection coils, thereby reducing the disturbance from the primary magnetic field and the environment. In comparison with the “coaxial two-coil” sensor [8, 9, 19] commonly used in MIPS detection technology, this sensor eliminated the disturbance from environmental magnetic field and the normal physiological changes in rabbits through the natural symmetry between the left and right brain hemispheres and the structural symmetry between the two detection coils. The phase detection also exhibited high sensitivity and strong antijamming capability.

On the basis of the MIPS technology, Gonzalez et al. [19] realized 24-h monitoring of acute focal cerebral ischemia in mice by using a “coaxial two-coil” sensor. The MIPS data first declined and then increased at approximately 10 MHz. With the help of the MIPS technology, Li et al. [9] conducted 24-h monitoring of epidural freezing-induced cerebral edema with a “coaxial two-coil” sensor. In comparison with the above mentioned, a “symmetric cancellation-type” magnetic induction sensor was used in the present study to realize 24-h monitoring of acute focal cerebral ischemia in rabbits on the basis of the MIPS technology. The trauma was smaller, and the animal model agreed with the clinical pathological development process of cerebral ischemic stroke to a greater extent. Data comparison between the experimental group and the control group and nonparametric two-independent-sample rank sum test results (*p* < 0.05) showed that the established monitoring system was feasible, with potential clinical application values.

The MIPS variation trends in the experimental group shown in Fig. 3 demonstrated that the MIPS data presented the firstly declining and then increasing trend, and it reached the minimum point at 8 h. In the research of Gonzalez et al. [19], the MIPS data presented a similar variation trend at 10 MHz. The MIPS variation trend in the experimental group may be related to the occurrence of cytotoxic edema and vasogenic edema after cerebral ischemia. According to the measurement result of Song et al. [27], the dielectric property presented a first increasing and then decreasing trend after focal cerebral ischemic injury in mice. As pointed out by Schafer et al. [28], the impedance of skeletal muscle also first increased and then decreased due to ischemia. According to the pathological process analysis of cerebral ischemic stroke, cytotoxic edema and vasogenic edema after cerebral ischemia constituted a dynamic variation process, where they played a dominant role at the early and later stages, respectively [27, 29].

Thus, in the present study, the MIPS variation trend in the experimental group corresponded to the pathological process of cerebral ischemic stroke. This phenomenon could be analyzed from the electrical characteristic changes caused by cytotoxic edema and vasogenic edema in the brain tissues. Previous research showed that the occurrence of cytotoxic edema is due to the failure of ionic pump or selective activation of ionic channel, further resulting in loss of steady-state ion gradient. Consequently, extracellular fluid enters the intracellular space [30, 31], which is then reduced [32]. The electrical conductivity of the brain tissues is also reduced [27], thus leading to a decline of MIPS in the experimental group. With the progression of cerebral ischemia, vasogenic edema is gradually developed due to BBB injury. Furthermore, because of perfusion of liquids, such as blood and cerebrospinal fluid, the amount of extracellular fluid increases [6], the electrical conductivity is elevated [27], and MIPS is increased in the experimental group. At this time, vasogenic edema plays a dominant role. The MIPS variation rate in Fig. 4 exhibited that the MIPS decline rate was the maximum at 5 h. This finding indicated that the development of cytotoxic edema was serious at this time; the subsequent decline rate started to decrease; and vasogenic edema started exerting its effect, played a dominant role at 9 h, and worsened at 16 h. Therapeutic methods could vary with the type of cerebral edema [33]. The above results indicated that the MIPS technology could provide useful information for treatment during the cerebral ischemia monitoring process.

## Conclusions

The MIPS technology integrates the merits of noninvasive, non-contact, and real-time continuous monitoring. The “thrombin induction method”, which conforms to the clinical pathological development process of cerebral ischemic stroke, was used in this study to establish an acute focal cerebral ischemia model in rabbits. A 24-h real-time continuous monitoring of acute focal cerebral ischemia in rabbits was realized through the MIPS technology by using a “symmetric cancellation-type” magnetic induction sensor with high phase detection sensitivity and strong antijamming capability. The experimental results proved that the MIPS technology-based monitoring system of acute focal cerebral ischemia in rabbits is feasible, with potential clinical application values. During the monitoring process of acute focal cerebral ischemia in rabbits, the MIPS technology showed a potential ability of differentiating the development process of cytotoxic edema from that of vasogenic edema, both of which are caused by cerebral ischemia.

## Methods

### Experimental animals and grouping

Healthy New Zealand white rabbits (provided by the Laboratory Animal Center of Army Medical University) weighing 2.0–2.5 kg were selected in this study. The random number table was used to divide the 22 rabbits, which were numbered as 1–22, into experimental group (10 rabbits), control group (5), and model verification group (7). Preoperative 24-h fasting was conducted with free access to water.

### Establishment of animal model

**Fig. 5:**
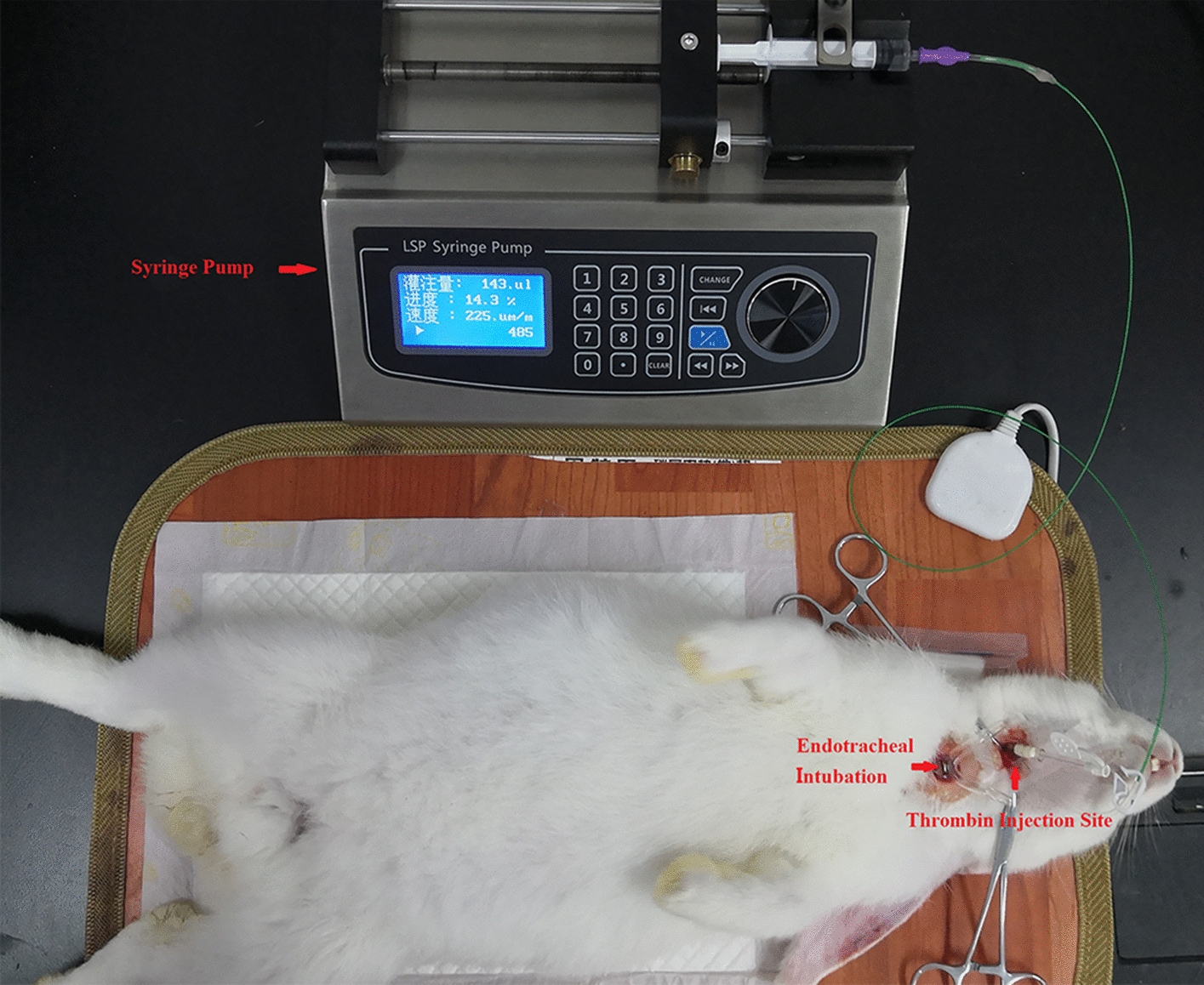
Thrombin injection process in rabbit

An acute focal cerebral ischemia model was constructed by injecting a thrombin mixture into the right CCA of each rabbit. Bovine thrombin (2908 NIH units/mg; Enzyme Research, South Bend, Indiana) was diluted to 666.67 NIH units/mL using normal saline, and rabbit brain thromboplastin (Neoplastine CI PLUS, Diagnostica Stago, Asnieres, France) was diluted to 0.2 mg/mL using normal saline. The diluted bovine thrombin and rabbit brain thromboplastin were mixed at 1:10 to prepare the thrombin mixture. A urethane solution (SCR, Shanghai, China) with a volume fraction of 25% was used for anesthetization; it was intravenously injected through the rabbit ear rim at a dose of 5 mL/kg. Meanwhile, 0.2 mL/kg of atropine sulfate (0.5 mg/mL; ELIPEX, Shanghai, China) was intramuscularly injected. After anesthetization, the hairs on rabbit head and neck were removed. Each rabbit was cut off from the middle of the neck, followed by blunt dissection of fascia and muscle. Tracheal intubation was conducted first, and then the exposed right CCA was separated. The bifurcation between the external carotid artery (ECA) and the internal carotid artery (ICA) was found to separate ECA and ICA. Before the thrombin mixture was injected, the ECA and ICA were first clipped. Subsequently, the indwelling needle was inserted above the ECA, reflexed at the bifurcation between the ICA and the ECA, and finally stretched deeply into the ICA. The thrombin mixture was slowly (25 μL/min) injected into the ICA with a syringe pump. After the injection was completed, the two ends of the injection point were ligated on the ECA, followed by wound suturing. Thrombin injection process in rabbit is shown in Fig. 5.

### Detection principle

**Fig. 6:**
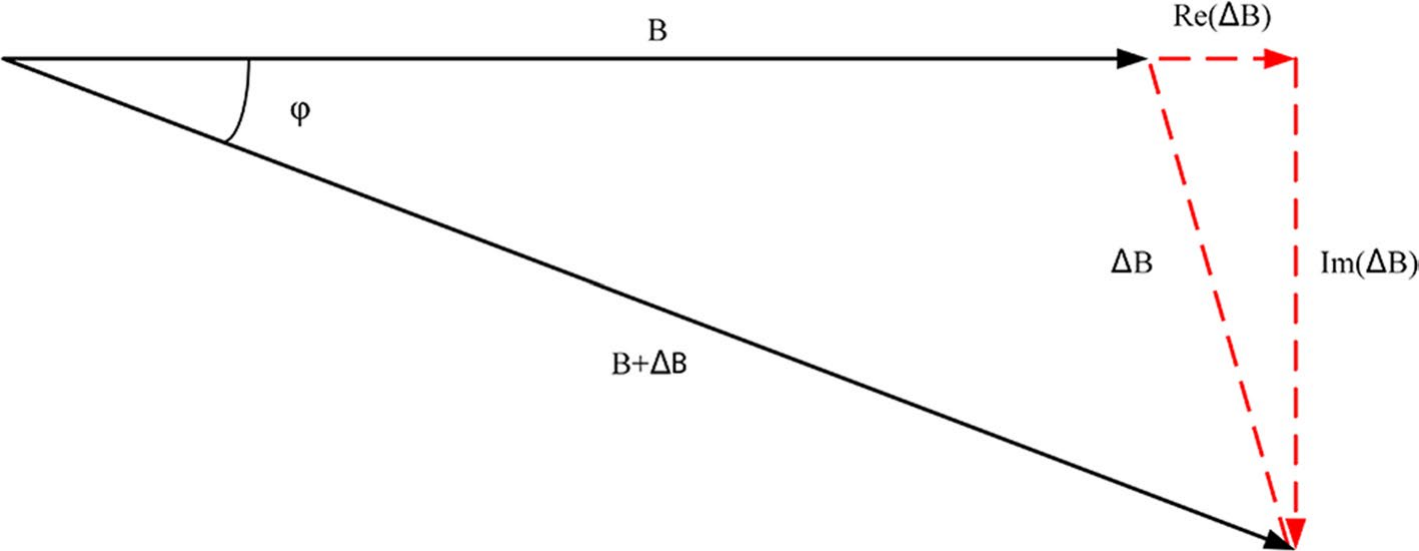
Vector graph of detection principle

The detection object was placed between the excitation coil and the detection coil, where the excitation coil was driven by an alternating current of a certain frequency to generate sinusoidal alternating primary magnetic field (B), which acted upon the object to generate eddy current and then form induced magnetic field (∆B), as shown in Fig. 6. If the excitation is conducted using sinusoidal alternating signal with angular frequency of $\omega$, the skin depth in the detection object is $\updelta ={(\frac{2}{\upomega {\mu }_{0}{\mu }_{r}\upsigma })}^{1/2}$, where $\upsigma$ and ${\mu }_{r}$ represent the electrical conductivity and the relative permeability of the detection object, respectively, and ${\mu }_{0}$ is the dielectric constant of free space. According to the research of Griffiths et al. [34], if the skin depth $\updelta$ of the electromagnetic field is much greater than the thickness of the detection object, ∆B and B present the following relationship:

1 $$\frac{\Delta \mathrm{B}}{\mathrm{B}}=\mathrm{Q\omega }{\mu }_{0}\left(\omega {\varepsilon }_{0}{\varepsilon }_{r}-i\sigma \right)+R\left({\mu }_{r}-1\right),$$

where ${\varepsilon }_{r}$ is the relative dielectric constant of the detection object, ${\varepsilon }_{0}$ is the dielectric constant of free space, and Q and R are geometric constants. Therefore, the induced current generated by the detection object generates an imaginary-part and negative component, that is, $\Delta \mathrm{B}$, which is in direct proportion to frequency and electrical conductivity. As the $\Delta \mathrm{B}$ value generated by biological tissues is much smaller than the B value and is usually determined by electrical conductivity, the following relationship exists:

2 $$\varphi \approx \left|\frac{\Delta \mathrm{B}}{\mathrm{B}}\right|\propto \omega \sigma .$$

Thus, the deflection angle $\varphi$ between the superposed magnetic field detected by the detection coil and the primary magnetic field is approximately in direct proportion to $\omega$ and $\sigma$.

## Experimental system

**Fig. 7.**
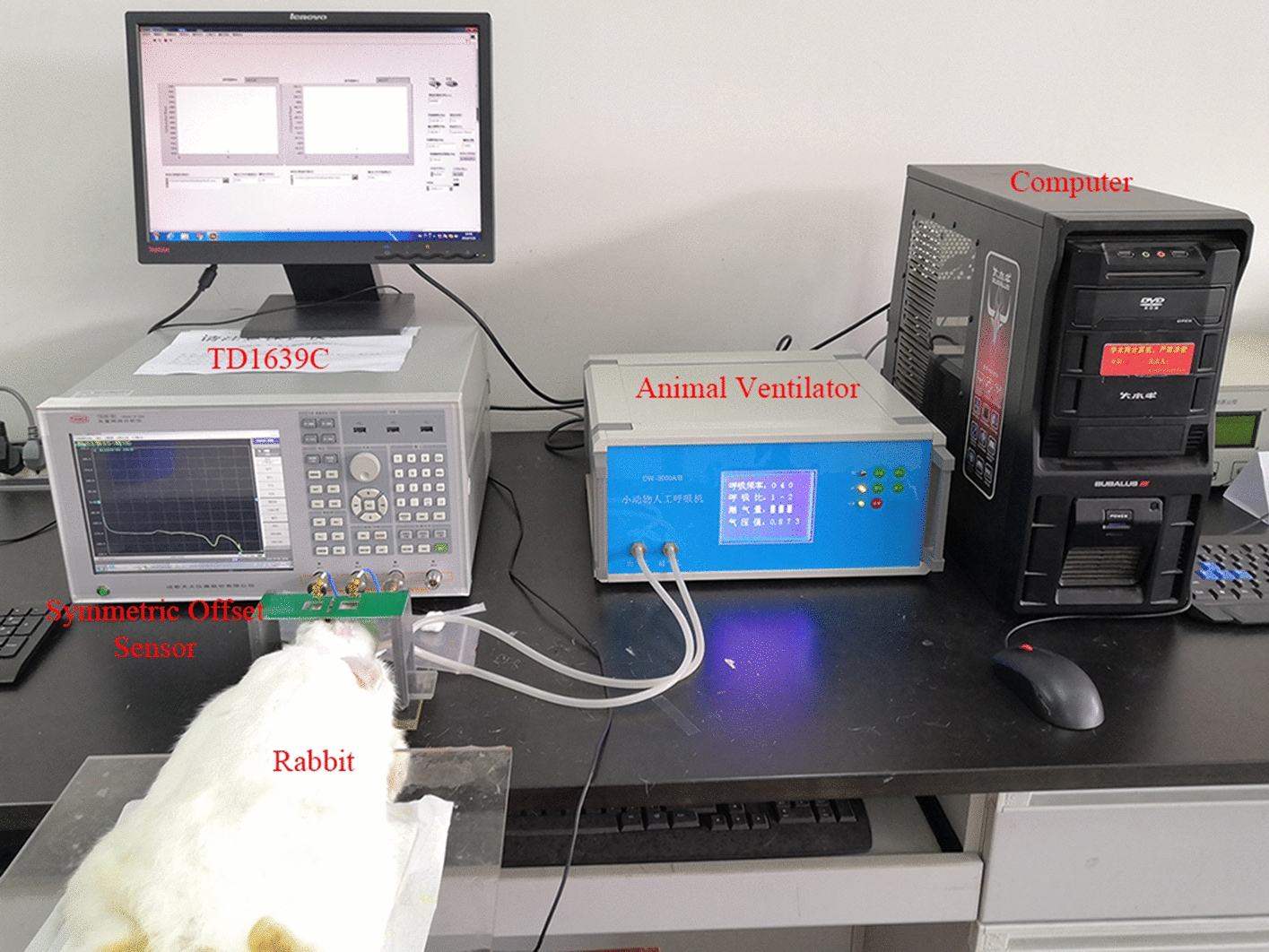
24-h monitoring system of acute focal cerebral ischemia in rabbits

The real-time monitoring system of acute focal cerebral ischemia in rabbits mainly included three parts: magnetic induction brain monitor, “symmetric cancellation-type” magnetic induction sensor, and animal ventilator (Fig. 7).

### “Symmetric cancellation-type” magnetic induction sensor

**Fig. 8:**
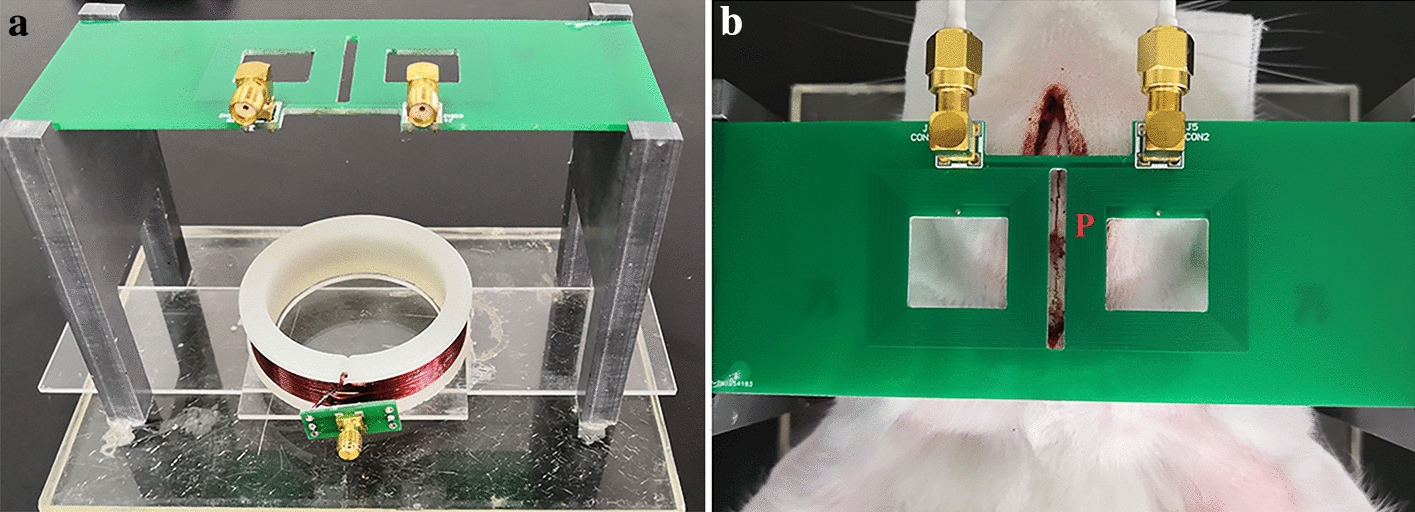
**a** “Symmetric cancellation-type” magnetic induction sensor and **b** relative positions of rabbit brain and sensor

The structure of the “symmetric cancellation-type” magnetic induction sensor, which consisted of one excitation coil and two detection coils, is shown in Fig. 8a. The rabbit was placed between the excitation coil and the detection coils. As the two brain hemispheres are relatively independent, one detection coil was placed above the normal brain hemisphere and the other one above the ischemic hemisphere. The two detection coils detected the primary magnetic field and induced the magnetic fields generated by both hemispheres. The primary magnetic fields detected by the two coils were the same, and the ischemic state was reflected by the phase difference between the induced magnetic field signals detected from both hemispheres. By virtue of natural symmetry of the left and right brain hemispheres and the structural symmetry of the two detection coils, this sensor could eliminate the disturbance from the normal physiological changes in rabbits and the environmental electromagnetic field and improve the detection sensitivity of the phase difference between magnetic induction signals in the left and right hemispheres.

The excitation coil was winded on an acrylic cylinder (diameter: 6.5 cm) with varnished wire (diameter: 0.8 mm), and the number of turns of the wire was 10. The detection coils were winded for 16 circles with a wire (diameter: 0.4 mm) to form a middle-hollow square with external side length of 29.2 mm and internal side length of 17.2 mm. The excitation coil and detection coils were fixed into a cube (length: 13.5 cm, width: 6 cm, and height: 11 cm) fitting the rabbit brain through the acrylic. The lower excitation coil could flexibly slide left or right for fine adjustment of geometric structural symmetry of the coil and reduce the difference of the primary magnetic field excited by the excitation coil between the two detection coils. Meanwhile, a sagittal suture of the rabbit brain was placed at the middle of the rectangular mouth (length: 29.2 mm, width: 0.3 mm) between the two detection coils, and the “cross stitch” of the rabbit brain was located at point P, as shown in Fig. 8b.

### Magnetic induction brain monitor

**Fig. 9:**
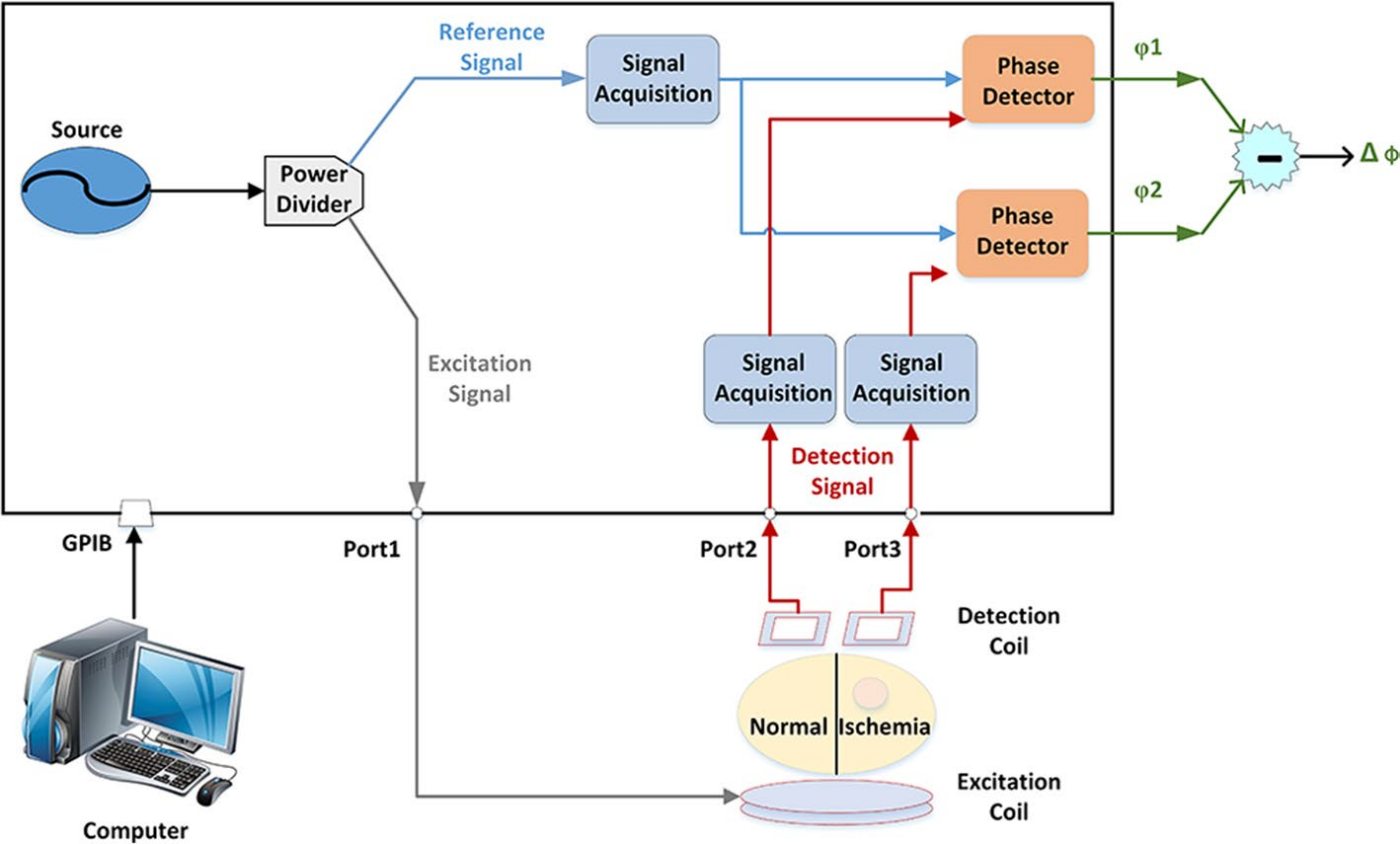
Flowchart of the experimental system

The magnetic induction brain monitor (TD1639C; TIANDA, Chengdu China) has various functions, such as output of excitation signal, phase detection, and data acquisition. As shown in Fig. 9, the signal source of the magnetic induction brain monitor outputted a signal, which was then divided by a power divider into excitation signal and reference signal. The excitation signal was outputted to the excitation coil via port 1 to generate the primary magnetic field. Then, it generated an induced magnetic field in the rabbit brain. The two detection coils received induced magnetic field and primary magnetic field from the left and right brain hemispheres, respectively, and the generated signals were acquired via port 2 and port 3. The detection signal of the two detection coils and reference signal passed two phase detectors, and the phase differences between detection signal and reference signal in the two coils were acquired, and referred to as ${\mathrm{\varphi }}_{1}$ and ${\mathrm{\varphi }}_{2}$. Therefore, the MIPS caused by focal cerebral ischemia is as below:

3 $$\mathrm{MIPS}=\Delta \mathrm{\varphi }={\mathrm{\varphi }}_{1}-{\mathrm{\varphi }}_{2}.$$

### Animal ventilator

The animal ventilator (DW-3000A/B; BRWL, Beijing, China) played an important role in this experiment. The respiratory frequency, tidal volume, and respiratory rate were set as 40 times/min, 20 mL, and 1:2, respectively, and tracheal cannula was connected for continuous ventilation. With the ventilator, the rabbit could be under normal cardiopulmonary functional state to increase the experimental success rate.

## Experimental method

### Model verification experiment

The brains of seven rabbits were taken at 0, 3, 6, 9, 12, 18, and 24 h after modeling, and 2% 2,3,5-triphenyltetrazolium chloride (TTC, Solarbio, Beijing, China) was used for staining. As a lipid-soluble photosensitive compound, TTC reacts with succinate hydrogenase to generate red formazan, but the dehydrogenase activity in ischemic tissues is degraded, thus failing to react with TTC. In addition, no change is generated and it presents a pale white color. Therefore, the cerebral infarction in rabbits could be reflected by color change.

### Experimental group

An acute focal cerebral ischemia model was first established for the rabbits in the experimental group, and the method was mentioned in Sect. 2.2. After the modeling ended, it was immediately placed in the magnetic induction brain monitor. The frequency band range, intermediate frequency bandwidth, and exciting power of this magnetic induction brain monitor were set as 100 kHz–100 MHz, 10 kHz, and 10 dbm, respectively. Afterward, the frequency point with the maximum transmission power was determined as the characteristic frequency point within the frequency band range. A 24-h real-time continuous monitoring was performed after parameter setting.

### Control group

The other steps conducted for the rabbits in the control group (n = 5) were the same as those conducted in the experimental group, except that thrombin mixture was not injected.

## Statistical approach

The means of all numerical values were expressed in the form of mean ± standard deviation. The differences between the experimental group and the control group were analyzed through nonparametric two-independent-sample rank sum test, and the feasibility of the monitoring system was verified from the statistical results. The nonparametric two-independent-sample rank sum test was performed to test the differences of MIPS data at initial time from those at 1 h and 2 h in the experimental group, so as to judge the time when thrombin exerted its function to induce cerebral ischemia. SPSS statistics software was used to conduct nonparametric two-independent-sample rank sum test, Mann–Whitney was selected for the test type. Finally, the Mann–Whitney U and Wilcoxon rank sum, Z-statistic and asymptotical significance (P) were output.

## Data Availability

All data generated or analyzed during this study are included in this published article.

## Abbreviations

BBB: Blood–brain barrier
CT: Computed tomography
MRI: Magnetic resonance imaging
ICP: Intracranial pressure
TCD: Transcranial Doppler sonography
EBI: Electrical bioimpedance
MIPS: Magnetic induction phase shift
CCA: Common carotid artery
ICA: Internal carotid artery
ECA: External carotid artery
MCA: Middle cerebral artery
TTC: 2,3,5-Triphenyltetrazolium chloride
